# Comparison of the Productivity of Primiparous Sows Housed in Individual Stalls and Group Housing Systems

**DOI:** 10.3390/ani10111940

**Published:** 2020-10-22

**Authors:** Yejin Min, Yohan Choi, Joeun Kim, Doowan Kim, Yongdae Jeong, Younghwa Kim, Minho Song, Hyunjung Jung

**Affiliations:** 1 Rural Development Administration, National Institute of Animal Science, Cheonan 31000, Korea; myjj0525@korea.kr (Y.M.); cyh6150@korea.kr (Y.C.); kjektw@korea.kr (J.K.); duwan38@korea.kr (D.K.); yongdaejeong@korea.kr (Y.J.); yhkims@korea.kr (Y.K.); 2 Division of Animal and Dairy Science, Chungnam National University, Daejeon 34134, Korea

**Keywords:** primiparous sows, group housing, stalls, welfare, productivity, reproductive performance

## Abstract

**Simple Summary:**

Recently, South Korea amended the respective laws and will be enforcing that sows must be kept in group housing after 6 weeks from insemination by the year 2030. Accordingly, the comparison of productivity of sows in individual stalls and group housing systems was investigated in order to provide information on group housing systems for sows to pig farms. Primiparous sows were divided into four groups and housed in equal number in pen stalls, in short stalls with non-gated feeding stalls, in free access stalls, or with access to electronic sow feeders after 8 weeks from artificial insemination. Sows were transferred to farrowing crates at 110 days of gestation. No differences were found in sow productive performance, reproductive performance, and colostrum composition among housing types. Therefore, it was concluded that group housing systems could be used to replace individual stalls in commercial sow units.

**Abstract:**

This study was conducted to provide commercial pig farms with information about group housing systems for sows in accordance with the amendment of the prohibition law for individual stalls for sows in South Korea. Therefore, this experiment was performed to compare the effects of individual stalls (IS) and group housing systems (GS) on the productivity of sows to investigate the feasibility of replacing individual stalls with group housing systems in commercial sow units. Forty primiparous sows (Landrace × Yorkshire; 210.67 ± 2.22 kg average initial body weight) were randomly assigned to four treatments with restricted feeding after 8 weeks from artificial insemination. The four treatments were (i) individual stalls (IS; housed in pen stalls), (ii) short stalls (SS; sows housed in pens with non-gated feeding stalls), (iii) free access stalls (FAS; a non-competitive housing system), and (iv) electronic sow feeders (ESF; used with radio frequency identification technology to allow individual sow management without individual confinement). All sows were transferred to farrowing crates at 110 days of gestation. There were no differences in sow productive performance, reproductive performance, and colostrum composition between IS and GS and among GS. The considered GS did not negatively affect any productivity parameters of primiparous sows compared with IS; the GS could replace IS in commercial sow units.

## 1. Introduction

There has been a transition from individual stalls (IS) to group housing (GS) in the swine industry owing to increased consumer pressure and animal welfare regulations and policies [1,2]. Although IS has been used to manage sows efficiently and minimize the fighting between sows, it is prohibited in many countries on the grounds of violating animal welfare [3,4]. The European Union in 2013 banned the use of IS for breeding sows, except for the first four weeks of pregnancy and the week before giving birth [5]. The United States has established guidelines for each state (including Florida, Maine, Rhode Island, Oregon, Arizona, California, Colorado, Michigan, Massachusetts, and Ohio) to restrict the use of IS for breeding sows. South Korea has amended the respective laws, enforcing that sows must be kept in group housing after six weeks from insemination by the year 2030 [6]. The Korea pig industry is gradually moving towards large-scale farming; however, there are still several active small-scale farms, which makes it difficult to obtain adequate information about the management of new facilities [7]. In addition, research about the farm animal welfare as well as group housing systems of sows was started only 10 years ago and information is still insufficient in South Korea. Understanding the effects of moving from IS to GS on changes in productivity of sows is essential in order to optimize animal welfare and increase the economic return from farms. Thus, there is an urgent need for research that investigates the effects of transitioning to GS from IS on the productivity of sows owing to the revisions in the Korean regulations pertaining to pig housing.

In regions such as Europe, where research on farm animal welfare is well established, various studies have been conducted to evaluate effects of group housing on productivity, welfare, management, and injuries of pregnant sows. According to a review article, animal welfare is affected by management procedures and facilities, including the group types (static and dynamic groups), floor space allowance, group size, floor quality, and feeding system [8,9]. Regarding sow reproduction, several studies have reported sows in GS had similar or improved reproductive performance compared with those in IS [10,11,12,13]. The findings of these studies may be a result of the poorer body condition of sows in IS, which could be a consequence of raised lower critical temperature [14,15], while activity increments could reduce farrowing time or birth intervals in GS [16,17,18].

The productivity of sows can change depending on the type of selected GS [10,11,18]. The commonly used GS include short stalls (SS), free access stalls (FAS), electronic sow feeders (ESF), and floor feeding (FF). The SS does not have gates; hence, sows can move freely; its length varies from the shoulder, hip, and overall length of the sow. In FAS, a gate is shut after a sow goes into a stall that accommodates only one sow; the sow leaves by opening the gate using her buttocks; thus, sows are not disturbed by other sows during feeding. The ESF can accommodate 30–40 sows with one electronic feeder, and the feed intake for each sow can be set using radio-frequency identification (RFID) technology. In this system, sows are not disturbed by other sows during feeding, while feed is placed on the flat floor in the FF system. Previous studies were performed to compare ESF and IS [10,11], but there is limited information about the comparison of sow productivity between IS and GS, excluding ESF. As many factors affect reproductive performance and welfare of sows in GS, there is a need to evaluate various formats of GS on sow reproduction. Therefore, the objective of this study was to compare the productivity of sows housed in IS and groups with SS, FAS, and ESF in order to optimize the conditions of housing sows under group conditions.

## 2. Materials and Methods

The experimental protocols for this research were reviewed and approved by the Institutional Animal Care and Use Committee at the National Institute of Animal Science (NIAS-2019-323).

### 2.1. Experimental Design and Animals

**Figure 1:**
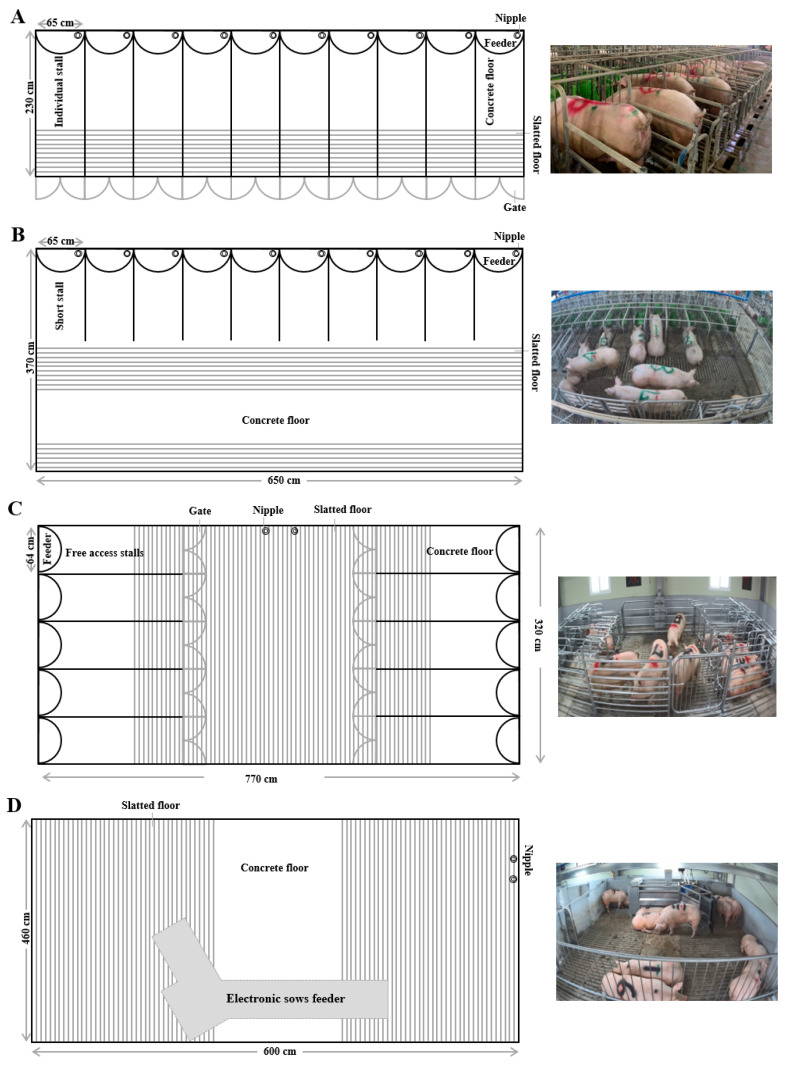
Types of group housing facilities of sows. (**A**) Individual stalls (IS). (**B**) Short stalls (SS). (**C**) Free access stalls (FAS). (**D**) Electronic sow feeders (ESF).

Forty primiparous sows (Landrace × Yorkshire; 210.67 ± 2.22 kg of average initial body weight (BW)) were randomly assigned to four treatments (IS, 1 sow/pen; three types of GS, 10 sows/pen) in a completely randomized design. The four treatments were (i) individual stalls (IS; sows housed in pen stalls, 0.65 × 2.3 m^2^, Figure 1A), (ii) short stalls (SS; sows housed in pens with non-gated feeding stalls, 0.65 × 3.7 m^2^, Figure 1B), (iii) free access stalls (FAS; non-competitive housing system; each sow occupies a stall with the gate closed, restricting other sows from interfering during feeding, after which sows exit the stalls backward using their buttocks, 3.2 × 7.7 m^2^, Figure 1C), and (iv) electronic sow feeders (one ESF (4 × 1.1 m^2^) was used with RFID technology that facilitates individual sow management without individual confinement, 4.6 × 6.0 m^2^, Figure 1D).

All sows were transferred to individual pens for 21 days prior to artificial insemination and randomly assigned to SS, FAS, and ESF, excluding IS after eight weeks from artificial insemination. Then, all sows were transferred to farrowing crates (1.8 × 2.4 m^2^) at 110 days of gestation. Sows were housed in GS with a partially slatted concrete floor and provided daily with a diet of approximately 2.0–2.5 kg/sow/day. The feed was provided in two meals a day at 09:00 and 16:30; individual feeder access was facilitated via ESF transponders. Water was offered to all sows ad libitum during the experimental period. Diets (Table 1) were formulated to meet or exceed the nutrient requirements for sows recommended by NRC (Nutrient Requirements of Swine of National Research Council, 2012). For the welfare of sows and piglets during the time of delivery, nursing was conducted for 24 h and hormonal drugs (Monzal^®^, Labiana Life Sciences, Barcelona, Spain) were administered for sows with dystocia symptoms (with a farrowing interval of more than 4 h). The lactation period was about 28 days and then sows returned to IS.

### 2.2. Measurements

#### 2.2.1. Sow and Reproductive Performance

Body weight (BW) and backfat thickness (BFT) of sows were measured at 50 days and 110 days of gestation and at 28 days of lactation. The BFT was measured at the P2 position (6 cm left and right from the center of the back of the 10th rib) using a medical imaging ultrasound (BF Ibex Pro, EI Medical Imaging, Loveland, CO, USA) [19].

All sows per treatment were observed using a video camera (HDR-AS50, Sony, Tokyo, Japan) for farrowing duration and farrowing interval. The farrowing duration was measured from the first birth to the last birth, and the farrowing interval was calculated by dividing the farrowing duration with the number of births. The estrus interval was recorded by observing sows twice a day (09:00 and 16:30) after weaning. Litter weight was measured within 6 h of farrowing and at 28 days of age. Survivability was calculated using pre-weaned and weaned piglet size after cross-fostering. The cross-fostering was evenly distributed according to the condition of the treated sows within 1 day after farrowing.

#### 2.2.2. Colostrum Composition

Colostrum samples for each sow were collected from the front two pairs of mammary glands using the hand massage method without chemical treatment after the birth of 3–4 piglets. Approximately 30 mL were collected in a 50 mL falcon tube (Milkoscan FT 120, Fourier-transform infrared spectroscopy, Hillerod, Denmark). The collected colostrum samples were stored in a freezer (−20 °C) until analysis. The component analysis was performed using the milk composition analyzer (CombiScope FTIR 300 HP, Delta Instruments, JB Drachten, The Netherlands) by thawing colostrum at about 20 °C. The IgG concentrations were analyzed by a porcine IgG ELISA kit (E101-104, Bethyl Laboratories, Montgomery, AL, USA) using a microplate reader (VersaMax, Molecular Devices, San Jose, CA, USA) per the manufacturer’s instructions.

### 2.3. Statistical Analysis

The data generated in the present study were analyzed using the PROC GLM procedure of SAS (version 9.4, SAS Inst. Inc., Cary, NC, USA). The experimental design was a completely randomized design. The experimental units were sow and litter. The statistical model for sow productivity included housing types as a fixed effect and initial body weight as a covariate. When significant differences were identified among the treatment means, they were separated using Tukey’s honestly significant difference test. Probability values < 0.05 were considered significant.

## 3. Results

### 3.1. Sow Performance

There were no significant differences in BW and BF at day 50 or day 107 or in weaning weight of sows between IS and GS (Table 2). Similarly, no significant differences were found on gestation length, farrowing duration, and weaning-to-estrus interval (WEI) of sows between IS and GS (Table 3).

### 3.2. Reproductive Performance

No significant differences were observed on litter size, litter weight, piglet weight, and average daily gain (ADG) and survivability of piglets between IS and GS (Table 4).

### 3.3. Colostrum Composition

The colostrum compositions were not significantly different between IS and GS (Table 5).

## 4. Discussion

The purpose of this experiment was to determine the feasibility of replacing IS with GS by comparing sow productivity under GS and IS regimes. Practical data supporting the similarity in the productivity of sows in IS and GS would contribute towards amending the South Korean animal welfare laws.

### 4.1. Sow Performance

In the pig industry, it is important to ensure that sows have an appropriate body condition to improve productivity; BW and BFT can be used as indicators of sows’ body condition [20,21]. In addition, BFT measurements can be used as a tool to evaluate animal welfare via the detection of sows with severe changes in body conditions [1]. In the present study, sows in the GS did not have different BW and BFT compared with those in IS. The results of the present study agree with those of Chapinal et al. [11], who observed no differences in BW and BFT for the sows housed in IS, SS, and ESF having a single feeder with no protection crate. A previous study by Zhao et al. [12] also reported similar results using IS and GS with a provided space of 2.5 m^2^ (per sow) in GS. In contrast, Kim et al. [22] reported that sows in GS with 3.5 m^2^ space per sow and individual feeders had greater BFT than those in IS. Estienne et al. [23] also observed higher sow body weight in GS than that in IS. These results may be because of the provision of the same amount of feed in a batch to sows in the IS and GS without adjustment in accordance with the body condition of the sows. As Estienne et al. [23] also reported, it may be related to lower muscle mass and bone strength due to the limited time of exercise of sows in IS compared with GS.

The present study showed that GS was not different for gestation length, farrowing duration, farrowing interval, and WEI of sows from IS. These results are consistent with those of Weng et al. [24], who reported that the farrowing duration was not different by IS, ESF, and GS with individual feeders. However, other studies showed that the farrowing duration was longer for sows housed in IS than GS [25,26]. Bates et al. [10] also reported that the WEI of sows housed in ESF was shorter than those housed in IS. Similarly, Kim et al. [22] observed that sows in GS with 3.5 m^2^ space (per sow) and individual feeders had shorter WEI than those in IS. These results may be related to the stress of sows due to different space allowances or housing types, such as GS and IS, that may cause physiological changes in sows, resulting in sow performance [27]. This diversity in research results mentioned above may be due to the differences in the duration of group housing, farm management, and exercise time based on the floor space allowance considered by the different studies.

### 4.2. Reproductive Performance

The present study showed that sows in GS did not have different litter size, litter weight, piglet size, piglet weight and ADG, and survivability of piglets compared with those in IS. These results agree with the results reported by Zhao et al. [12] and Zhou et al. [13], who observed no differences on litter size, litter weight, and piglet weight and ADG between IS and GS. In contrast, Bates et al. [10] reported that sows housed in ESF had higher litter and piglet weights than those housed in IS. McGlone et al. [28] also reported that the reproductive performance of sows in GS was better than or similar to that in IS.

According to a review paper, the embryos are attached to the uterine wall for approximately 11–16 days after fertilization, explaining why sows should avoid stress for 2–4 weeks after insemination [8]. However, the present study showed the transfer of sows to GS after eight weeks from artificial insemination did not negatively affect sow reproductive performance. Moreover, the regulations in most countries have been legislated to ensure that the sows are moved to GS four weeks before farrowing. These results may indicate GS can replace IS successfully in the near future.

### 4.3. Colostrum Composition

Colostrum functions as a major factor in improving the passive immunity and metabolic energy of piglets [29,30]. There have been very few studies about the colostrum components in GS sows; colostrum components have been often thought to be affected by environmental stressors during pregnancy [30]. The present study found no differences on total solids, protein, fat, lactose, and IgG in colostrum of sows housed in IS and GS. Zhou et al. [13] reported a similar finding, which showed no difference in the protein, triglycerides, triiodothyronine, thyroxine, and prolactin in the colostrum of sows housed in IS or 1.2 m^2^ and 2.5 m^2^ spaces in GS. In addition, Zhao et al. [12] found no significant differences in the composition of plasma IgG and IgM for sows housed in IS and GS. Thus, it is suggested that GS did not negatively affect the physiological properties of sows to induce changes in the colostrum composition compared with IS.

## 5. Conclusions

Recently, South Korea has amended the respective laws and will be enforcing that sows must be kept in group housing after 6 weeks from insemination by the year 2030. Accordingly, the comparison of productivity of sows in individual stalls and group housing systems was investigated in order to provide information on group housing systems for sows to pig farms. As a result, there were no differences on sow productive performance, reproductive performance, and colostrum composition between IS and GS and among GS. This study confirmed that IS could be substituted by GS. However, this conclusion may be limited because this study was conducted on a well-controlled experimental farm with a small number of sows. Therefore, due to the revision of the Korean law, additional research is necessary on various aspects such as the workload for each GS, investment costs, number of sows, impact on GS according to the parity of sows, and sow management in order to provide guidelines to farmers to successfully transition to GS.

## Figures and Tables

**Table 1:** Composition of the experimental diets (as-fed, pelleted form).

Ingredients, %	Gestation	Lactation
Corn	58.80	59.47
Lupine seed	6.00	-
Wheat bran	11.00	8.00
Soybean hull	4.00	-
Soybean meal	9.00	21.10
Rapeseed meal	3.00	3.00
Animal fat	2.50	3.60
Molasses	1.80	0.50
L-Lysine	0.33	0.57
Threonine	0.02	0.15
Tryptophan	0.10	0.05
Monodicalcium phosphate	1.50	1.20
Limestone	1.38	1.67
Salt	0.40	0.40
Vitamin and Mineral Premix ^1^	0.15	0.15
Phytase	0.02	0.02
Total	100.00	100.00
**Analyzed Chemical Composition, %**		
DE (kcal/kg)	3300.00	3480.00
CP	14.31	17.31
Ca	0.93	0.96
P	0.67	0.64
Lys	0.79	1.05
Met	0.22	0.33
Thr	0.53	0.78

^1^ Supplied per kilogram diet: vitamin A, 9600.00 IU; vitamin D_3_, 1800.00 IU; vitamin E, 24 mg; vitamin K_3_, 1.5 mg; vitamin B_1_, 1.5 mg; vitamin B_2_, 12 mg; vitamin B_6_, 2.4 mg; vitamin B_12_, 0.045 mg; pantothenic acid, 24 mg; niacin, 45 mg; biotin, 0.09 mg; folic acid, 0.39 mg. Supplied per kilogram diet: Fe, 150 mg; Cu, 96 mg; Zn, 72 mg; Mn, 46.5 mg; I, 0.9 mg; Se, 0.3 mg.

**Table 2:** Effects of individual stalls (IS) and group housing systems (GS) on sow performance.

Items ^1^	IS	GS			SEM ^2^	*p* Value
		SS	FAS	ESF		
Number of sows	10	10	10	10	-	-
BW, kg						
Gestation						
At d 50	210.63	211.22	211.40	212.19	2.22	0.977
At d 107	242.97	242.86	239.31	242.24	2.85	0.804
Change, +	32.34	31.64	27.91	30.05	3.06	0.789
Lactation						
At weaning	208.23	216.26	204.11	207.47	3.74	0.162
Change, −	34.74	26.60	35.20	34.77	4.48	0.481
BFT, mm						
Gestation						
At d 50	22.08	20.80	22.22	21.00	0.78	0.419
At d 107	23.08	22.10	24.72	23.89	1.12	0.397
Change	1.00	1.30	2.50	2.89	0.91	0.448
Lactation						
At weaning	19.83	17.25	19.33	18.89	1.17	0.409
Change, −	3.25	4.85	5.39	5.00	0.79	0.412

^1^ SS, short stall; FAS, free access stall; ESF, Electronic sow feeders; BW, body weight; BFT, backfat thickness. ^2^ SEM, standard error of means.

**Table 3:** Effects of individual stalls (IS) and group housing systems (GS) on gestation length, farrowing duration, and weaning-to-estrus interval (WEI) of sows.

Items ^1^	IS	GS			SEM ^2^	*p* Value
		SS	FAS	ESF		
Number of sows	10	10	10	10	-	-
Gestation length, d	115.8	115.0	115.4	115.2	0.37	0.449
Farrowing duration, h	5.14	4.84	4.06	6.41	0.68	0.192
Farrowing interval, min	28.32	34.72	24.81	30.20	5.93	0.582
WEI, d	5.93	5.81	5.11	5.50	0.34	0.374

^1^ SS, short stall; FAS, free access stall; ESF, Electronic sow feeders. ^2^ SEM, standard error of means.

**Table 4:** Effects of individual stalls (IS) and group housing systems (GS) on sow reproductive performance.

Items ^1^	IS	GS			SEM ^2^	*p* Value
		SS	FAS	ESF		
Litter size, no. of piglets						
Total born	12.00	10.80	11.33	13.11	1.00	0.423
Stillbirth	1.00	0.40	1.33	1.44	0.50	0.479
Mummy	0.22	-	0.22	0.22	0.11	0.477
Dead ^3^	0.11	0.20	0.22	0.67	0.21	0.341
Born alive	10.89	10.10	9.78	11.00	1.03	0.810
Cross-fostering	11.00	10.10	9.78	11.00	0.56	0.334
Weaned	10.00	9.70	9.11	10.44	0.64	0.540
Litter weight, kg						
Total born	17.31	16.17	17.94	18.40	1.28	0.644
Born alive	15.60	15.22	15.54	15.44	1.25	0.997
Cross-fostering	82.77	82.59	76.53	82.83	4.64	0.743
Weaned	82.77	82.59	76.53	82.83	4.64	0.743
Piglet weight, kg						
Total born	1.50	1.54	1.63	1.42	0.08	0.373
Born alive	1.49	1.54	1.65	1.43	0.08	0.268
Cross-fostering	1.47	1.51	1.60	1.42	0.08	0.428
Weaned	8.49	8.56	8.46	8.00	0.31	0.586
Average daily gain, g/pig ^4^	273.10	278.65	268.12	260.04	10.74	0.650
Survivability, % ^4^	91.03	96.16	92.94	94.88	3.56	0.768

^1^ SS, short stall; FAS, free access stall; ESF, Electronic sow feeders. ^2^ SEM, standard error of means. ^3^ Number of the dead piglets after farrowing for 1 day. ^4^ Data represent piglet performance from cross-fostering to weaning.

**Table 5:** Effects of individual stalls (IS) and group housing systems (GS) on colostrum composition of sows.

Items ^1^	IS	GS			SEM ^2^	*p* Value
		SS	FAS	ESF		
Number of sows	10	10	10	10	-	-
Total solids, %	24.99	25.88	25.91	27.14	0.77	0.287
Protein, %	15.63	15.70	16.55	15.15	0.80	0.694
Fat, %	5.58	6.58	5.77	6.53	0.67	0.633
Lactose, %	2.84	2.83	2.62	2.71	0.11	0.472
IgG, μg/mL	285.01	263.23	289.64	207.72	59.20	0.784

^1^ SS, short stall; FAS, free access stall; ESF, Electronic sow feeders. ^2^ SEM, standard error of means.
